# Protein Methylation in Diabetic Kidney Disease

**DOI:** 10.3389/fmed.2022.736006

**Published:** 2022-05-12

**Authors:** Ye Cheng, Yanna Chen, Guodong Wang, Pei Liu, Guiling Xie, Huan Jing, Hongtao Chen, Youlin Fan, Min Wang, Jun Zhou

**Affiliations:** ^1^Department of Anesthesiology, The Third Affiliated Hospital of Southern Medical University, Guangzhou, China; ^2^Department of Anesthesiology, The Eighth People’s Hospital of Guangzhou, Guangzhou, China; ^3^Department of Anesthesiology, Guangzhou Panyu Central Hospital of Panyu District, Guangzhou, China; ^4^Department of Anesthesiology, The Gaoming People’s Hospital, Foshan, China

**Keywords:** chronic kidney disease, renal fibrosis, protein methylation, histone methylation, nonhistone methylation

## Abstract

Chronic kidney disease (CKD) is defined by persistent urine aberrations, structural abnormalities, or impaired excretory renal function. Diabetes is the leading cause of CKD. Their common pathological manifestation is renal fibrosis. Approximately half of all patients with type 2 diabetes and one-third with type 1 diabetes will develop CKD. However, renal fibrosis mechanisms are still poorly understood, especially post-transcriptional and epigenetic regulation. And an unmet need remains for innovative treatment strategies for preventing, arresting, treating, and reversing diabetic kidney disease (DKD). People believe that protein methylation, including histone and non-histone, is an essential type of post-translational modification (PTM). However, prevalent reviews mainly focus on the causes such as DNA methylation. This review will take insights into the protein part. Furthermore, by emphasizing the close relationship between protein methylation and DKD, we will summarize the clinical research status and foresee the application prospect of protein methyltransferase (PMT) inhibitors in DKD treatment. In a nutshell, our review will contribute to a more profound understanding of DKD’s molecular mechanism and inspire people to dig into this field.

## Introduction

Chronic kidney disease (CKD) is a fatal cause of mortality worldwide. The prevalence of CKD has increased steadily over the past decade. Besides the increased mortality, CKD also significantly lessens patients’ quality of life and imposes a financial burden on the economy (1–4). CKD is diagnosed when there is a chronic reduction in kidney function and chronic damage in the structure. Both events are the final common pathological manifestations of renal fibrosis. Renal fibrosis represents unsuccessful wound healing of the kidney tissue. This fibrosis is characterized by glomerulosclerosis, tubular atrophy, and interstitial fibrosis. As fibrosis evolves, an increasing number of nephrons lose their function (5, 6).

Approximately half of all patients with type 2 diabetes and one-third with type 1 diabetes will develop CKD, which is clinically defined by the presence of impaired renal function or elevated urinary albumin excretion or both (7, 8). DKD has been traditionally viewed as a microvascular disorder, clustered along with retinopathy and neuropathy, and separate from the macrovascular disease that contributes to coronary heart disease (CHD), peripheral vascular disease, and cerebrovascular disease. However, each disorder can be considered tissue-specific manifestations of the same pathogenetic process. DKD is the renal manifestation of the same glucose-driven process at susceptible sites elsewhere in the body. Although all cells are chronically exposed to high plasma glucose levels in diabetic patients, only some manifest progressive dysfunction. The endothelial cells lining the vasculature are a prime example. Specifically, the inability of endothelial cells to down-regulate their glucose transport in response to high glucose leads to an overwhelming flux of intracellular glucose, which triggers the generation of pathogenetic mediators that contribute to the development of diabetic complications, including DKD (9, 10).

Despite genetic risks, environmental influence may be strongly associated with susceptibility to DKD. Epigenetic modifications refer to gene transcription changes that manifest in the phenotype, which can be inherited through multiple cell divisions. As the most pervasive epigenetic modifications, DNA methylation and histone modifications cause stable gene expression *via* chromatin remodeling. These mechanisms play a role in determining the developmental cell fate and human physiological and pathological processes. As the most intensively investigated PTM, protein methylation has become a prominent research topic of interest. Protein methylation is part of many critical biological functions that play a role in healthy physiological development and diseases, such as obesity and type 2 diabetes (11).

In this review, to present existing knowledge on the underlying mechanism of protein methylation, we consider the biological function of protein methylation, followed by the relationship between protein methylation and DKD. The evidence presented here supports the expectation that drugs targeting protein methylation will prove value for DKD treatment.

## Biological Functions of Protein Methylation

Protein methylation, including histone methylation and non-histone protein methylation, is an essential type of PTM (12). Protein methylation mainly occurs on lysine and arginine. The methylation is performed by protein lysine methyltransferases (PKMTs) and protein arginine methyltransferases (PRMTs), including members of the PRMT family, SET gene family, and non-SET gene family (13–15). Methylation occurs at different amino acid sites and in various forms. For example, lysine can be monomethylated (me1), dimethylated (me2), or trimethylated (me3), while arginine can be monomethylated (me1), symmetrically dimethylated (me2s), or asymmetrically dimethylated (me2a) on its guanidine group (16). These results indicate that protein methylation is flexible and diverse (17). Methylation affects protein activity, protein-protein interactions, and interaction with other PTMs (17).

**FIGURE 1:**
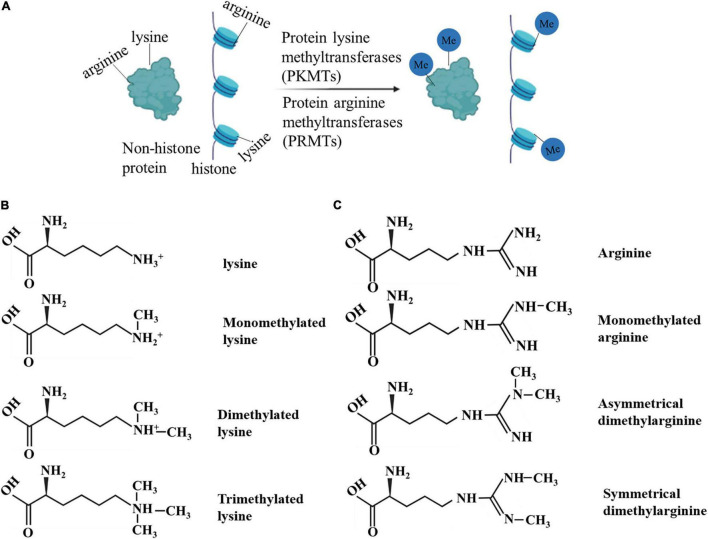
**(A–C)** Diverse protein methylation.

Histones are widely studied owing to their abundance and relatively easy detection. Protein methylation is often associated with histones and their roles in gene regulation (18). Histone methylation is associated with gene repression or activation, depending on which residue is modified (19). Methylation of histone H3 at lysines 4 or 36 positively correlates with the transcription rate of RNA polymerase (pol) II. Histone methyltransferases (HMTs) for these methyl groups interact with elongated RNA pol II to form histone methylations in transcribed regions. In contrast, transcription repression factors recruit repressive HMTs for H3K9 or H3K27 methylation (20–24). Lysine and arginine methylation have been found in hundreds of non-histone proteins, and the terms, PKMT and PRMT, are now more frequently used than the original term, HMT. In addition to histone-centric roles, the crucial role of non-histone proteins should not be ignored. The latter have significant implications for human health and the treatment of human diseases (25, 26) (Figure 1).

## Protein Methylation in Diabetic Kidney Disease

The associations between the dysregulation of PMT and the progression of many human diseases have been described (16, 27–29). SET9-mediated methylation of inhibitory Smad7, a crucial regulator of transforming growth factor-beta (TGF-β)/bone morphogenetic protein signaling by negative feedback loops, might lead to its degradation, thereby affecting TGF-β-dependent activation of extracellular matrix (ECM) genes. Critical components of mitogen-activated protein kinase, such as redundant acronym syndrome and retinoblastoma protein, are subject to lysine and arginine methylation, often in a complex manner (30, 31). Numerous studies have already revealed that protein methylation is associated with DKD and that the final common pathway in the transition from CKD to ESRD is fibrosis (6, 32). It is still in its infancy to understand protein methylation in DKD, including its occurence, progression, and treatment. However, research on the connection between histone methylation and non-histone methylation has flourished recently (33). Below, we summarize studies on the relationship between DKD and protein methylation, including histone methylation and non-histone methylation.

## Histone Methylation in Diabetic Kidney Disease

Methylation is the most widely studied modification of histone proteins. Methylation of histone proteins changes the transcription machinery, creating an active or repressive chromatin structure and transcriptional marks (34). In fact, histone methylation is usually considered a prevalent modification among core histone tails and is one of the most stable PTMs. Histone methylation mainly includes the methylation of lysine and arginine residues. Among these, lysine is the most well-studied. Histone methylation is dynamic and reversible. Histone methylation has a mixed gene expression effect, unlike histone acetylation linked to an “open” chromatin state and gene expression activation. Therefore, they can be marks of the active and repressive states of chromatin, thereby producing different developmental effects. Researchers have found that histone methylation plays an essential role in DKD progression (35, 36).

### Histone Lysine Methylation in Diabetic Kidney Disease

Different modified-residue of histone lysine corresponds to gene repression or activation. Methylation at H3K4, H3K36, and H3K79 correlates with gene transcriptional activation, whereas methylation at H3K9, H3K27, and H4K20 is related to transcriptional repression (37). The burgeoning research in this field has included investigations of the biological and pathological functions of histone methylation in some common diseases, including diabetic nephropathy (DN), one of the most common CKDs. Below, we focus on the role of histone lysine methylation in the development and progression of DN based on active and repressive chromatin marks (38).

#### Active Marks of Histone Lysine Methylation in Diabetic Kidney Disease

H3K4me1/2/3, H3K36me2/3, and H3K79me2 are associated with transcriptionally active regions. In addition, increasing evidence suggests that histone methylation regulates ECM and inflammatory genes in almost all renal cell types related to the pathogenesis of DKD (29, 39).

Transforming growth factor-beta 1-induced expression of ECM genes plays an important part in the development of chronic renal diseases, such as DN. Although many key transcription factors are studied clearly, it remains unclear how the modulation of nuclear chromatin influences the expression of ECM gene. Epigenetic chromatin marks, such as H3Kme, might play a role in TGF-β1-induced gene expression in rat mesangial cells (RMC) under normal and high-glucose (HG) conditions. When treated with HG and TGF-β1, the levels of H3K4me marks (H3K4me1/2/3) at their respective promoters are increased (40, 41). The gene expression levels of collagen 1alpha1 (Col1α1), plasminogen activator inhibitor 1 (PAI-1), and connective tissue growth factor (CTGF) in RMC are also upregulated. Metabolic memory existing in vascular dysfunction has been associated with H3K4me modification (42). One critical study reported the significance of alterations in histone methylation (specifically in the lysine residues) in acute kidney injury (AKI). H3K4me3 at pro-inflammatory genes (monocyte chemotactic protein 1 [MCP-1] and tumor necrosis factor-alpha [TNF-α]) and profibrotic genes (TGF-β1 and collagen III) were increased in renal ischemia reperfusion injury (IRI) animal models (43, 44). The TNF-α and MCP-1 genes characteristically showed H3K4m3 methylation in mouse models of AKI induced by IRI, endotoxin, unilateral ureteral obstruction, and maleate (43, 45, 46). In another study on patients with AKI, the levels of H3K4m3 at exon 1 of the hydroxy methylglutaryl-CoA (HMG-CoA) reductase genes were also upregulated (47). The upregulation of histone H3K4 me3 may be involved in podocyte dysfunction, which is the most common cause of primary nephrotic syndrome in the middle-aged and elderly. Collectively, the results indicate that H3K4 methylation is essential in the progression of DKD in diverse aspects, including both inflammatory and oxidant stress, all of which will result in renal fibrosis.

H3K36me3 is a chromatin marker associated with transcriptional elongation (24, 48). The levels of H3K36me3 were found to be higher at the monocyte chemoattractant protein 1 (MCP-1) and RAGE loci, and in the plasminogen activator inhibitor-1 (PAI-1) gene in db/db mice treated with water. After treatment with losartan for 10 weeks, the H3K36me3 levels decreased at the RAGE and PAI-1 loci (49). These changes imply the role of H3K36me in DN progression. Unlike most methylated sites located in the histone H3 tail, the H3K79 methylated site is situated in the histone globular domain. Methylation of H3K79 catalyzed by a disruptor of telomeric silencing-1H3K79me plays an essential role in cell cycle regulation, embryonic development, DNA damage response, hematopoiesis, cardiac function, and development of leukemia (37). One report described the involvement of the dynamic regulation of H3K79me in fluid reabsorption, which is essential for blood pressure control and electrolyte homeostasis in kidney collecting ducts. The downregulation of H3K79me at the epithelial sodium channel promoter can lead to increased gene expression in response to aldosterone signaling (50–52), and decreased H3K79me2 may contribute to the changes in DN patients and mouse cortical collecting duct M1 cell models (53). These results suggest that H3K36me and H3K79me may play a crucial part in fluid reabsorption and chronic changes of kidney structure, which might severely affect the normal function of the kidney.

#### Repressive Marks of Histone Methylation in Diabetic Kidney Disease

H3K9me2/3, H3K27me3, and H4K20me3 are generally associated with gene silencing or repression. Histone methylation may be responsible for the “metabolic memory” phenomenon that leads to long-term changes in diabetic complications, including DN, and plays essential role in the progression of KD in fibrotic, inflammatory, and oxidative stress pathways.

Based on multiple evidence, H3K9me plays a crucial role in the development of DN. HG can stimulate decreased H3K9me3 levels at the promoters of critical inflammatory genes, such as interleukin (IL)-6, macrophage colony-stimulating factor, and MCP-1. These events increase the expression of these inflammatory genes in normal human vascular smooth muscle cells (VSMCs). Similar chromatin lysine methylation changes were demonstrated in the VSMCs of db/db mice compared to non-diabetic control db/ + mice (54). TNF-α induction can also lead to sustained decreases in H3K9me3 at promoters following increased inflammatory gene expression in the VSMC of db/db mice (51, 55). Of note, a similar change was noted in RMC models following treatment with TGF-β and HG. The reduced levels of the repressive marks, H3K9me2 (di-methylation at the 9th lysine residue of the histone H3 protein) and H3K9me3, at their respective promoters (40, 49) can induce the upregulation of the Col1α1, PAI-1, and CTGF genes.

**FIGURE 2:**
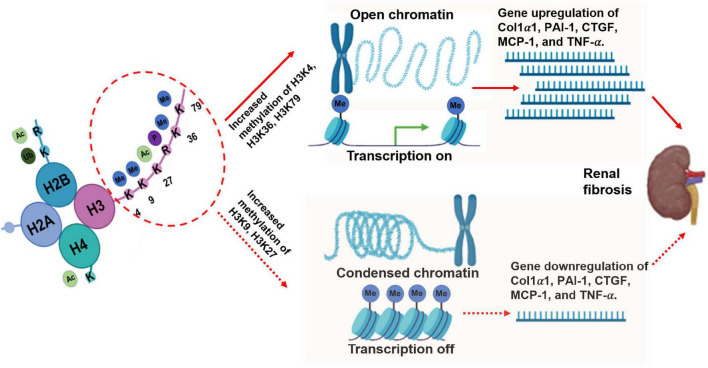
Histone lysine methylation in DKD.

H3K27me is associated with gene repression (56, 57). H3K27me3 levels at PAI-1 promoters were reportedly decreased in an animal model of type 2 diabetes. In another study, cyclo-oxygenase-2 (COX2) and MCP-1 were upregulated in the kidneys of OVE26 mice. The OVE26 mouse is characterized by transgenic overexpression of calmodulin in pancreatic β cells, deficient insulin production, and type I diabetes. This phenomenon has been associated with decreased H3K27me3 levels and H3K27me3 demethylase KDM6A/UTX) (58). Altogether, these findings regarding H3K27me3 further imply the role of histone methylation in DKD (Figure 2).

### Histone Arginine Methylation in Diabetic Kidney Disease

The methylation of arginine residues by PRMTs is involved in regulating basic cellular processes, including DNA damage response, RNA transcription and processing, signal transduction cascades, and liquid-liquid phase separation. Recent evidence has considerably advanced the identification of clinically relevant PRMT inhibitors (59) based on the defined physiological roles of PRMTs. The inhibitors have been linked to cancer, metabolic diseases, neurodegenerative disorders, and some kidney diseases (15, 60), thereby enabling the development of experimental tools.

Asymmetric dimethylarginine (ADMA) is a naturally occurring dimethylated analog of the amino acid. ADMA inhibits nitric oxide synthase and is an established cardiovascular risk factor in many diseases, including CKD (61–63). Atherosclerotic cardiovascular disease is the leading cause of death in patients with CKD. However, the underlying vascular disease mechanisms are not fully elucidated. Altered arginine methylation is associated with the degree of atherosclerosis in a CKD mouse model, suggesting the therapeutic potential of interrupting this pathway in CKD-related atherosclerosis (64). Indeed, elevated plasma levels of ADMA have been observed in patients with hypertension and CKD (61, 65–67). Furthermore, Andrade et al. observed that patients who received a renal transplant displayed only moderately decreased renal function but presented disturbances in the methylation cycle and arginine-creatine pathway that lead to increased plasma values of homocysteine (Hcys), S-adenosylhomocysteine, and ADMA. Defective methylation also contributes to endothelial dysfunction due to the impaired production of NO, which has been observed in patients that received a renal transplants (68). Further investigation of the possible benefits of appropriate therapeutic measures is warranted (69).

## Non-Histone Protein Methylation in Diabetic Kidney Disease

Although some methylating enzymes are histone-specific, many have now been found to modify both histone and non-histone substrates (70–72). Non-histone protein methylation has recently emerged as a PTM with wide-ranging cellular functions (73–76). The methylation of non-histones plays a significant part in the pathogenesis of DKD. SMYD2 is a SET and MYND domain-containing HMT that methylates histone and non-histone proteins. For example, the tumor suppressors RB, p53, and the molecular chaperone heat shock protein 90 have been identified in the pathogenesis of autosomal dominant polycystic kidney disease (ADPKD) (77–81). The transcriptional regulators, STAT3 and p65, are SMYD2 non-histone substrates. Upregulation of SMYD2 leads to methylation of STAT3 at lysine 685, methylation of p65 at lysine 310, and partial methylation at lysine 221, followed by their phosphorylation and activation to regulate proliferation and apoptosis of renal epithelial cells (82). SMYD2 is overexpressed in cystic renal epithelial cells, it might serve as a promising target for novel ADPKD therapies (83). The enormous potential of drugs that target protein methylation will likely only be realized if their effects on non-histone protein methylation are better understood.

## Protein Methylation as New Directions for the Diagnosis and Treatment of Diabetic Kidney Disease

**FIGURE 3:**
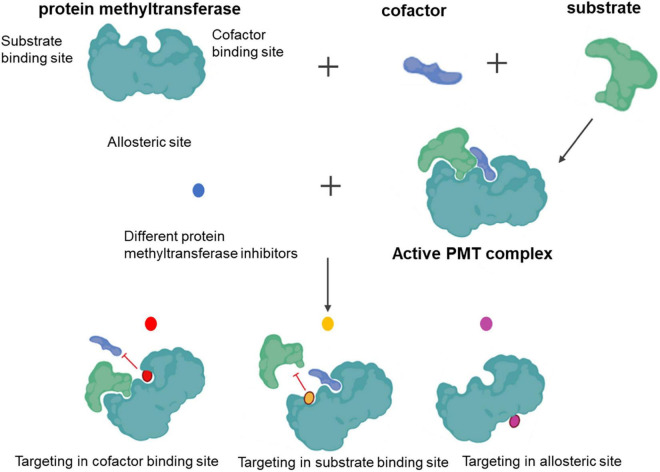
Functional mechanism of protein methyltransferase inhibitors.

All PMTs share a common catalytic mechanism. The SAM donor and peptide methyl-acceptor bind to different but connected surfaces within the active site to form a functional ternary complex. Following the assembly of the complex, direct transfer of the methyl group from SAM to the substrate proceeds *via* a classical bimolecular nucleophilic substitution (SN2) reaction. Chemical inhibition targets either cofactor or substrate-binding sites, as well as by allosteric means (Figure 3).

Knowledge of the pharmacological modulation of proteins that write, read, and erase methyl marks is needed (84, 85). We have witnessed advances in PMT inhibitors from tool compounds to precision medicines. Indeed, several histone methyltransferase inhibitors have already reached clinical application (86), demonstrating the availability of PMTs as a target class. In addition to histone-centric roles, PMTs are also vital for regulating non-histone proteins, with significant implications for human health and disease treatment (25, 26). Well-defined sets and selective chemical probes have provided valuable tools for investigating biological mechanisms. Panels of well-validated protein methyltransferase probes with demonstrated utility in interrogating complex biological systems have also been described (87). Selective, potent, and cell-active inhibitors of both lysine and arginine methyltransferases have been developed by exploiting the cofactor-binding site, substrate peptide-binding site, and, less commonly, distal allosteric pockets. In 2007, the first selective small-molecule inhibitor of PKMT, BIX-01294, was reported (88). Since its discovery, this inhibitor has been used to probe G9a involvement in cellular reprogramming (89, 90). Unfortunately, the poor separation between cytotoxic and functional effects has limited the broader utility and adoption of this compound. The discovery of UNC0638 primarily overcame this issue. UNC0638 can reduce the abundance of H3K9me2 and can reactivate G9a-silenced genes and a retroviral reporter in mouse embryonic stem cells, demonstrating its usefulness in studying the biology of G9a/glucagon-like peptides (91). Although numerous studies have linked G9a to disease (92, 93), no G9a inhibitors are currently used in the clinic. The five SMYD lysine methyltransferase members methylate both histone and non-histone proteins (94). Although limited SMYD2 inhibitors have been reported, we believe that its application prospects are vast.

Renal fibrosis is the common pathological hallmark of chronic kidney disease, and the SET domain-containing lysine methyltransferase 7 (SETD7) promotes renal fibrosis considerably. Ice treated with PFI-2, an inhibitor of SETD7, presented less bone marrow-derived myofibroblasts, fewer CD206 + /α-smooth muscle actin + cells, and developed less renal fibrosis. Furthermore, SETD7 inhibition reduced the infiltration of inflammatory cells and decreased the production of pro-inflammatory cytokines and chemokines in the kidneys after folic acid treatment (95). Some *in vivo* and *in vitro* studies demonstrate that emodin reduced extracellular collagen deposition and inhibited Smad3 and CTGF pro-fibrotic signaling pathways, which were correlated with the down-regulation of EZH2 and reduced trimethylation of histone H3 on lysine 27 (H3k27me3) in NRK-49F fibrotic cells and UUO kidneys (96). Disruptor of telomeric silencing-1 like (DOT1L) protein specifically catalyzes the methylation of histone H3 on Lys79 (H3K79) and is implicated in tumors. DOT1L inhibition increased expression of phosphatase and tensin homolog, a protein associated with dephosphorylation of tyrosine kinase receptors, and prevented the decline in levels of Klotho and Smad7, two renoprotective factors. Targeting DOT1L attenuates renal fibrosis by inhibiting renal fibroblasts and EMT by suppressing the activation of multiple profibrotic signaling pathways while retaining the expression of renoprotective factors (97).

From a therapeutic point of view, reader antagonism may provide alternative routes to modulate methyl signaling pathways in DKD, which may be far-reaching when resistance has developed to existing clinical candidates (98, 99). Recent advances in the development of methyl-lysine reader antagonists present opportunities to selectively intervene in the downstream signaling of the methyl mark or as alternative sites to target PMTs themselves (100). In addition, individual proteins often contain several distinct reader modules with different binding capabilities (101). Potent, selective, and cell-active antagonists of reader function are, therefore, valuable tools to decipher the individual contributions of distinct reader domains in addition to uncovering potential therapeutic value. For example, a-366 was recently shown to antagonize recognition of H3K4me3 by the Tudor domain of Spindin1, a methyl-lysine reader (IC50 = 182.6 ± 9.1 nM). Although significant progress has been made, much remains to be learned about the pharmacology and biology of most PMTs (99, 102). Furthermore, such knowledge is expected to considerably advance CKD treatment.

## Conclusion and Future Perspectives

Both histone methylation and non-histone methylation are associated with various renal diseases. Moreover, they participate in regulating ECM and inflammatory genes in almost all renal cell types (19, 103). The search for the link between protein methylation and DKD mainly focuses on histones, especially histone lysine methylations (104, 105). Notably, histones can be marks of the active, “poised,” and repressive chromatin states. An increasing number of PMTs could serve as new epigenetic therapy agents for multiple diseases, including DKD, in the future (106, 107). In addition to studying protein methylation, the cross-interaction among DNA methylation, protein methylation, and protein acetylation is an essential area of study regarding the pathogenesis, development, and progression of DKD. Further efforts are urgently needed and should not be limited to a single layer.

## Author Contributions

YeC and GW: writing – original draft preparation. GX, PL, and HJ: writing – review and editing. YaC and MW: visualization. HC, YF, and JZ: funding acquisition.

## Conflict of Interest

The authors declare that the research was conducted in the absence of any commercial or financial relationships that could be construed as a potential conflict of interest.

## Publisher’s Note

All claims expressed in this article are solely those of the authors and do not necessarily represent those of their affiliated organizations, or those of the publisher, the editors and the reviewers. Any product that may be evaluated in this article, or claim that may be made by its manufacturer, is not guaranteed or endorsed by the publisher.
